# Lei Wei (Ed.): Advanced fiber sensing technologies

**DOI:** 10.1007/s00216-021-03761-2

**Published:** 2021-11-24

**Authors:** Lothar Leidner

**Affiliations:** Group of Optical Spectroscopy, Institute of Theoretical and Physical Chemistry, University of Tuebingen, Auf der Morgenstelle 18, 72076 Tübingen, Germany


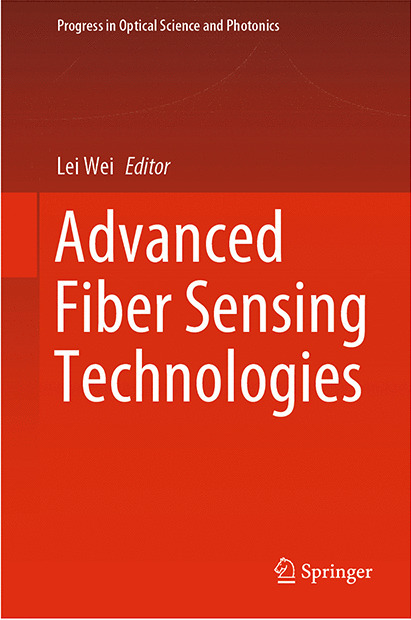


**Bibliography**

Advanced fiber sensing technologies

Lei Wei (Ed.)

Series: Progress in Optical Science and Photonics

ISBN: 978-981-15-5506-0

Springer

Hardcover, 303 pages

2020

Optical sensors play a significant role in medicine, environmental monitoring, process control, and many other applications. They are small and immune to electromagnetic interference, and do not conduct electricity. They can be designed to withstand high temperatures. Fiber optic sensors have the additional advantage of a high level of integration, which allows even smaller sensor sizes to be realized, and the absence of light coupling issues. Due to their flexibility, they can be used under harsh conditions and in hard-to-reach places. The book introduces the complex world of multi-functional multi-material fibers. Theory, design, and application as well as future trends are discussed. Practical fiber devices with optoelectronics, photonics, acoustics, biomedicine, and energy harvesting functionalities are presented.

## Contents

The book is part of a series called Progress in Optical Science and Photonics. It is divided into fifteen independent chapters, all dealing with issues related to fiber sensing technologies with the primary aim to improve sensor performance. Beyond that, it is difficult to find a common denominator.

Topics are advanced fiber structures, such as micro-structured fibers or fibers, tapered to wavelength scale diameters, as well as new fiber materials. Well-known optical sensing principles, such as SPR, LSPR, SERS, optical gratings, and interferometers, are integrated into fibers.

New and revived production techniques are presented: structuring of materials using polymer cold drawing process; thermoelectric flexible fibers; “in-fiber breakup” technique to modify structures and hereby to achieve in-fiber material engineering for sensing applications; sapphire-derived fibers working under harsh environment conditions.

With regard to wearable devices, and fiber-based energy harvesting and storing, thermoelectric materials are used as a new type of flexible fibers.

## Comparison with the existing literature

There are two newer books which cover a similar subject: *Optical fiber sensors: advanced techniques and applications*, edited by Ginu Rajan (CRC Press), and *Fiber optic sensors*, written by Shizhuo Yin, Paul B. Ruffin, and Francis T.S. Yu (CRC Press). Both books present design, operation, and practical applications of fiber optic sensing systems including many kinds of sensing and medical applications.

A third monograph, treating the concept of wearable electronics, is the title *Flexible and wearable electronics for smart clothing - aimed to smart clothing*, edited by Chengyi Hou, Hongzhi Wang, and Gang Wang (Wiley). In this book, fiber and textile related thermo-electrics, functionalization of fiber materials for washable smart wearable textiles, and textile triboelectric nano-generators for energy harvesting are presented.

## Critical assessment

The individual chapters are written independently of each other by different authors. So there is no need to read the book from front to back. All chapters are structured in the same way: Abstract, Keywords, Introduction, Main Body Text, Conclusion, and References. Some authors go without renaming the Main Body Text into something more specific (subject related). There are many acronyms in the book that are widely known to the expert. Nevertheless, it would be helpful to provide the reader with a corresponding list to look them up.

In the book, many sophisticated and highly ambitious techniques are presented. The terminus “cutting-edge advances” stated by the publisher is not an exaggeration. With some topics, you need a lot of optimism to believe in success. Nevertheless, the demonstrated will to succeed is impressive and contagious. Certainly, some of the visions will lead to new insights and solutions.

## Readership recommendation

As stated by the publisher, the intended audience are scientists of the related fields and advanced graduate students. I agree to this assessment.

## Summary

*Advanced fiber sensing technologies* offers an overview of the state-of-the-art in the fields of design, specific materials, and structures of fiber sensors and numerous applications. With wearable electronic devices in mind, energy harvesting and storage are also considered.

